# Congenital Mastocytosis: Case Report and Review of the Literature

**DOI:** 10.7759/cureus.10565

**Published:** 2020-09-21

**Authors:** Lacie Turnbull, Darlene A Calhoun, Vibhuti Agarwal, Dennis Drehner, Caroline Chua

**Affiliations:** 1 Orthopaedics and Rehabilitation, University of Florida, Gainesville, USA; 2 Pediatrics, Nemours Children's Hospital, Orlando, USA; 3 Pathology, Nemours Children's Hospital, Orlando, USA

**Keywords:** newborn rash, diffuse cutaneous mastocytosis, mast cells, flushing, biopsy

## Abstract

Mastocytosis is a rare infiltrative disorder characterized by mast cell proliferation within the skin and various extra-cutaneous organ systems. We report the case of a full-term neonate admitted to the neonatal intensive care unit for evaluation of diffuse skin lesions on her face, trunk and extremities. Initially, the lesions appeared to be consistent with a blueberry muffin rash. However, over a period of days the lesions became vesicular and changed in shape and number. The neonate underwent evaluation for infective etiologies, skin biopsy of the lesions, and flow cytometry analysis of the peripheral blood. The surgical pathology examination of the skin biopsy demonstrated mast cells consistent with a diagnosis of cutaneous mastocytosis. A review of relevant literature is also provided.

## Introduction

Mastocytosis is a rare and clinically heterogeneous disease characterized by abnormal accumulation of mast cells in various tissues. It can be limited to the skin as in cutaneous mastocytosis, or it can involve the bone marrow, liver, spleen, and lymphatic tissues as in systemic mastocytosis. The age of development is bimodal, with most pediatric patients exhibiting symptoms before the age of two years and adults developing the disorder after the age of 15 years [1]. There are three major types of cutaneous mastocytosis: diffuse cutaneous mastocytosis (DCM), urticaria pigmentosa (UP), and solitary mastocytoma. DCM is the least common and the most severe form, characterized as a generalized erythroderma with a reddish to brown-orange discoloration and extensive bullae. Blisters may be present in 62-69% of cases and become hemorrhagic with predominance on the trunk and extremities [2]. Patients also commonly present with pruritus, urticaria, a positive Darier's sign, and marked dermatographism. With the release of mast cells, these patients also exhibit flushing, hypotension, severe anaphylaxis, hepatomegaly, diarrhea, and gastrointestinal (GI) bleeding [2]. The accumulation of mast cells in the dermis of cutaneous mastocytosis results in maculopapular lesions commonly mistaken for a variety of pediatric rashes and closely resembles the characteristic rash of blueberry muffin syndrome. Diagnosis of cutaneous mastocytosis is based on clinical findings and skin biopsy [1,2]. The neonatal presentation of DCM is extremely rare. This case report describes a case of congenital DCM in a full-term neonate.

## Case presentation

**Figure 1:**
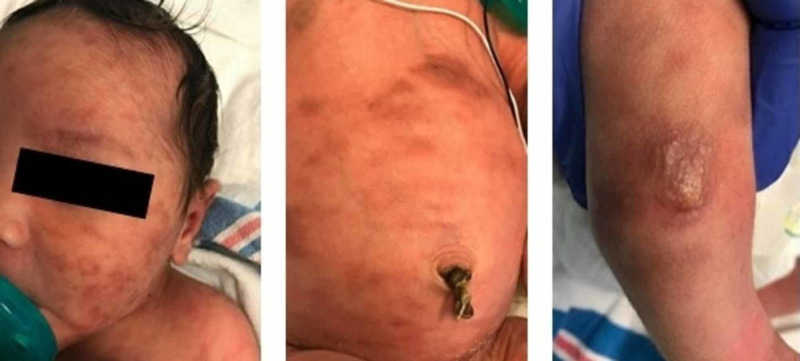
Photos of neonate with diffuse cutaneous lesions Facial (Left panel), abdomen (Middle panel), and extremity (Right panel) lesions.

A female neonate born at 39 weeks and one day, weighing 3458 grams, was delivered by spontaneous vaginal delivery to a 19-year-old Gravida 1 mother who had received limited prenatal care. The pregnancy was complicated by oligohydramnios. Prenatal labs were unremarkable. Specifically, the mother had negative assays for syphilis, rubella, hepatitis B infection, human immunodeficiency virus, herpes simplex virus (HSV), chlamydia, and gonorrhea. Artificial rupture of the membranes occurred four hours prior to delivery with terminal meconium noted. The neonate was noted to have skin lesions that resembled a “blueberry muffin” appearance characterized as scattered purpuric papules, macules, and bullae on the scalp, face, neck, chest, abdomen, and extremities, but no mucosal involvement (Figure 1).

The rest of the physical examination was normal. The neonate was non-dysmorphic, well-appearing, with normal vital signs, and without evidence of lymphadenopathy or hepatosplenomegaly.

A sepsis evaluation was performed, including blood and fungal cultures and blood/surface HSV polymerase chain reaction. The neonate received empiric therapy consisting of ampicillin, gentamicin, fluconazole, and acyclovir. Additional viral test results for rubella, parvovirus, varicella, and toxoplasma were all negative.

Laboratory investigations revealed mild leukocytosis, lymphocytosis, mild neutropenia, normal hemoglobin, and platelets (Table 1). Peripheral blood flow cytometry was negative for neoplastic myeloid B or T lineage population, including leukemia.

**Table 1:** Complete blood count

Hematologic Parameters	Patient’s Values	Ref Range and Units
WBC	11.2	7.46-14.55 K/UL
Hemoglobin	16.9	12.7-14.9 G/DL
Platelet	277	150-400 K/UL
Neutrophils	11.8	21.2-55.4%
Lymphocytes	71.4	8-46%
Absolute neutrophils	1.3	3.91-8.26 K/UL
Absolute lymphocytes	8	1.46-3.78 K/UL

**Figure 2:**
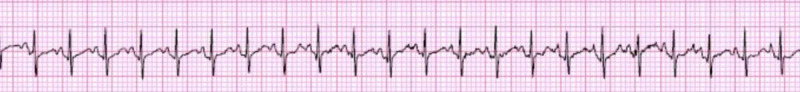
Electrocardiogram Sinus tachycardia

The neonate was noted to have self-limited episodes of tachycardia associated with redness and flushing of her skin lasting 15-30 minutes with heart rates as high as 220 bpm. Although the neonate was hemodynamically stable, she appeared to be uncomfortable as exhibited by flexion of both thighs which improved after the passage of flatus. An electrocardiogram showed sinus tachycardia (Figure 2), and echocardiogram revealed a tiny patent foramen ovale and peripheral pulmonic stenosis which resolve within one year of age. Ultrasound of the abdomen was negative for any visible lesions/masses.

**Figure 3:**
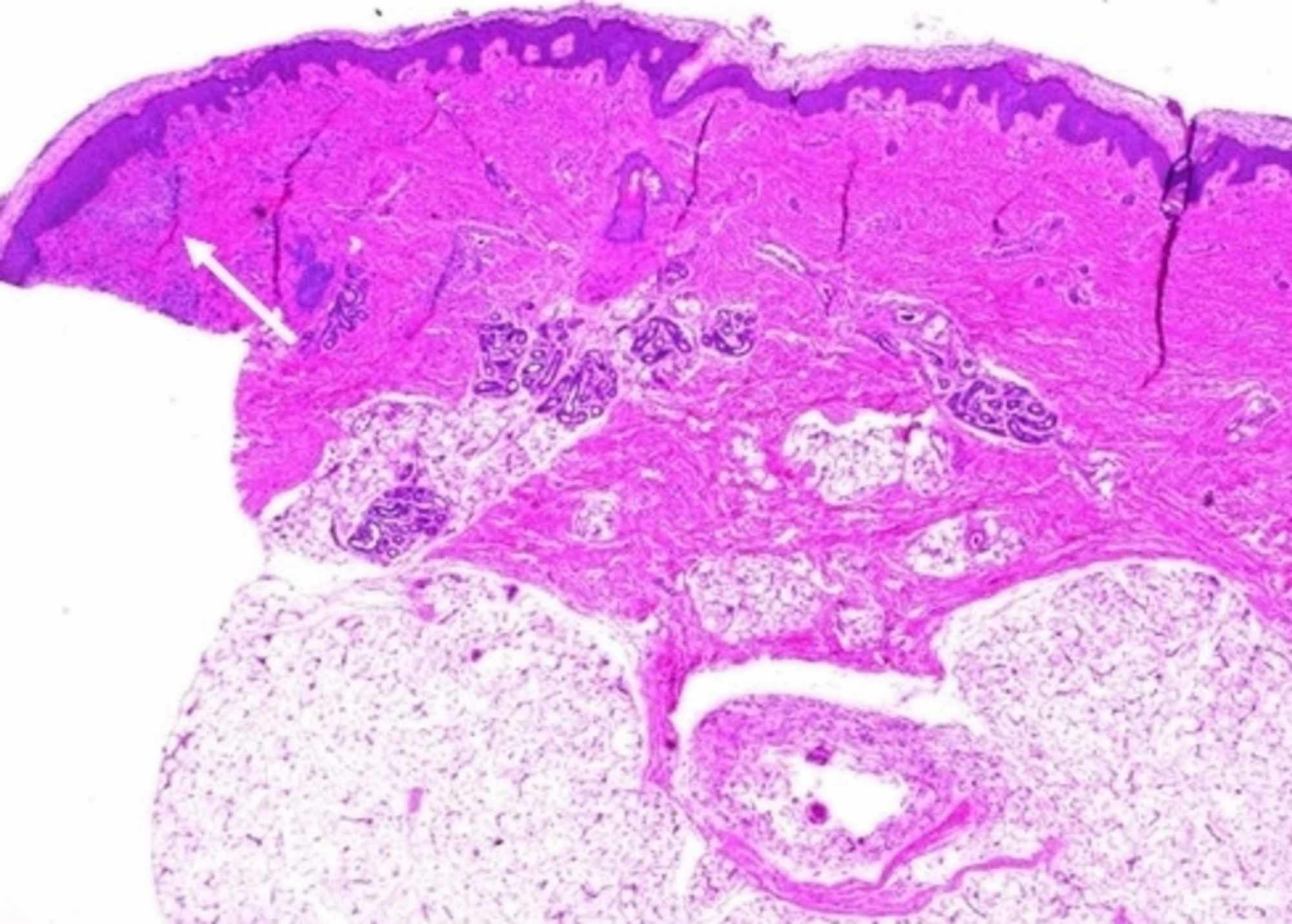
Low power view of skin lesion Low power view (40X) showing a moderately dense mast cell infiltrate in the upper dermis (white arrow).

**Figure 4:**
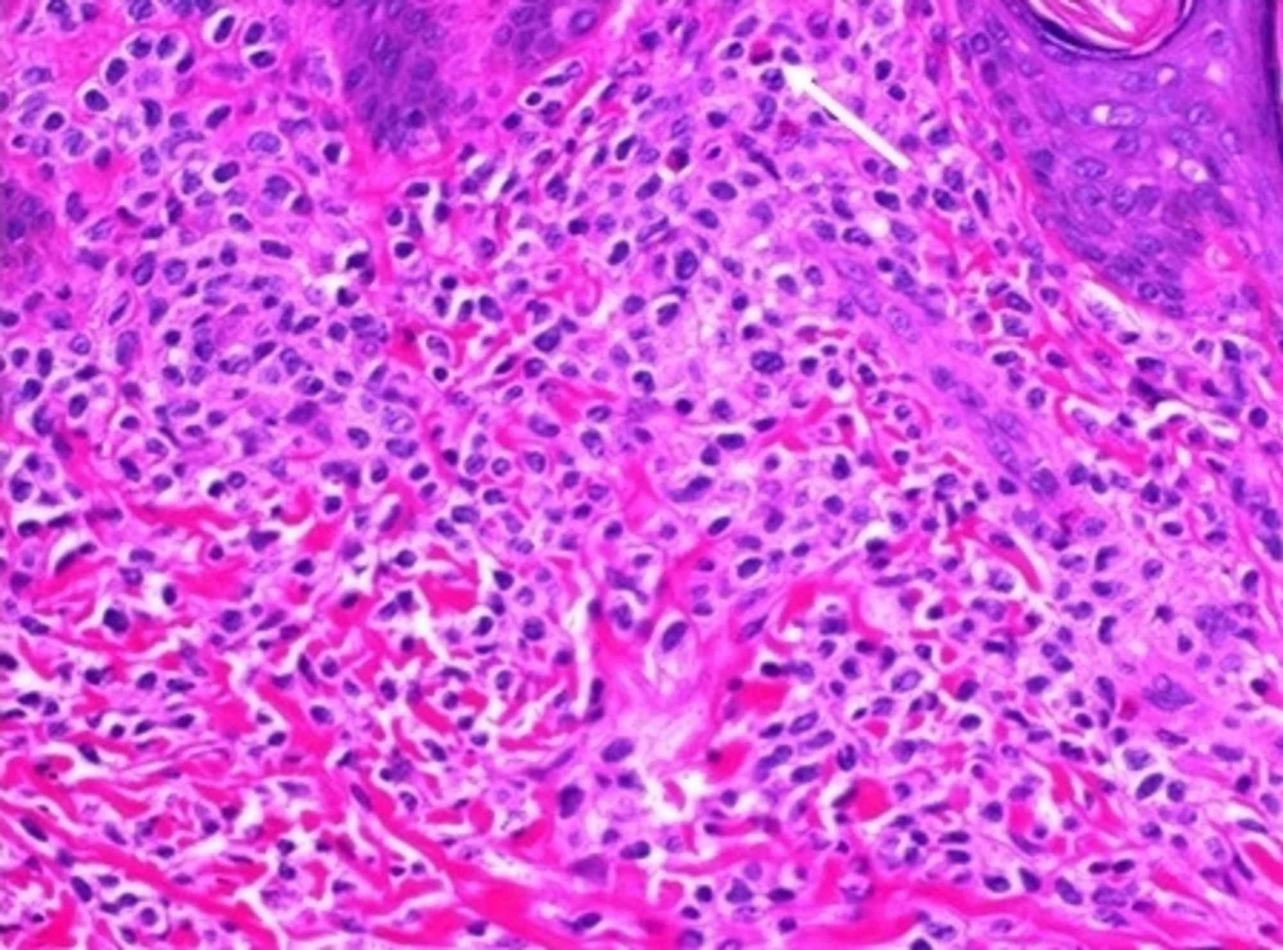
High power view of skin lesion High power view (400X) showing monomorphic infiltrate with interspersed eosinophil (white arrow).

Skin biopsy of two lesions was performed by the pediatric surgeon on day seven of life; one biopsy was from a regressing lesion on the lower extremity and the other from the edge of an active lesion on the mid-back. The former showed a minimally dense chronic inflammatory cell infiltrates in the dermis. In the latter, at one edge of the biopsy, there was a mild to moderately dense infiltrate of monotonous mononuclear cells. Occasional eosinophils were scattered among the mononuclear cells. Immunohistochemical assays for CD117, CD45, CD68, and CD33 were 2-3+/4 positive in the infiltrate. A Giemsa stain showed eosinophilic granules in the cells, and mast cell tryptase was 1-2+/4 positive in the cells of the infiltrate (Figures 3, 4).

The diagnosis of DCM was made.

The neonate was able to orally feed without any issues and was hemodynamically stable throughout the remainder of her hospital stay. She was discharged to home on day 15 of life in a stable condition with follow-up visits with pediatric hematology and pediatric rheumatology. The serum tryptase, a marker for mast cell activation, was 34.6 ng/mL (normal: less than 11.5 ng/mL) at six weeks of age and increased to 53.8 ng/mL by 3.5 months of age. Serum-specific IgG testing to common food was negative. The c-KIT Asp816Val mutation analysis, a measurement of mast cell burden and disease aggressiveness, performed at 3.5 months of age was negative. The infant is being treated with H1-blocker (hydroxyzine), H2-blocker (famotidine), a mast cell stabilizer (cromolyn sodium), and received a short course of oral corticosteroid (prednisolone) with a decrease in the skin lesions and the frequency of flushing episodes.

## Discussion

Recognition of this rare form of DCM in a neonate is important, as it can be easily mistaken for other newborn rashes. Additionally, it can be associated with life‐threatening complications including anaphylaxis, GI bleeding, and secondary bacterial infections leading to septic shock and death [3]. The blueberry muffin appearing rash in neonatal patients often presents a diagnostic challenge with a broad range of differential diagnoses, including infections (cytomegalovirus, rubella, toxoplasmosis, HSV, coxsackie, parvovirus, Epstein-Barr virus, syphilis); blood cell dyscrasias (ABO and Rh incompatibility, hereditary spherocytosis, twin-twin transfusion syndrome); and hematologic or other malignancies (congenital leukemia, Langerhans cell histiocytosis, neuroblastoma, mastocytosis) [4]. The misdiagnosis of DCM is not uncommon and can lead to delayed treatment for these patients.

In addition to laboratory evaluation for infectious etiologies, it is essential to obtain a biopsy early on in the diagnostic workup of these patients. A study by Stewart et al. reported a similar presentation of cutaneous mastocytosis in which the patient’s presentation and history were suspicious for an infectious etiology and similar to our patient, received a complete sepsis work-up. The patient had two attempted lumbar punctures and was treated with broad-spectrum antimicrobials. It was not until a biopsy was performed that cutaneous mastocytosis was revealed [5].

Selection of sites for skin biopsy of diffuse processes is fraught with the possibility of sampling error. In this case, the biopsy from the regressing lesion had non-specific changes. In the diagnostic biopsy, the infiltrate was present in approximately 15% of the specimen. The appearance of the cells and the presence of eosinophils in the infiltrate raised the possibility of Langerhans cell histiocytosis. However, CD1a, a specific marker for Langerhans histiocytosis [6], was negative. Leukemia cutis was another diagnostic consideration and includes myeloid leukemia with monocytic/histiocytic differentiation being the most frequent cause. CD45, CD33, CD68, and CD117 were positive. CD45 is expressed by most hematopoietic cells and excluded non-hematopoietic causes such as neuroblastoma [6]. CD33 and CD68 are expressed by myeloid lineage cells and cells with monocytic/histiocytic differentiation [6,7]. CD117 is expressed in myeloid stem cells and mast cells [6]. Mast cells are myeloid lineage cells and can express those markers. On initial evaluation, a Giemsa stain showed staining consistent with mast cells. Mast cell tryptase, a more specific mast cell marker [1], had faint positivity. Repeat mast cell tryptase at another institution showed positive staining consistent with cutaneous mastocytosis, further supporting the diagnosis of DCM. Tryptase levels exceeding 20 ng/mL are seen in mastocytosis [8]. At six weeks of age, the patient's serum tryptase was 34.6 ng/mL.

The main clinical features that led us to suspect mastocytosis was the generalized episodic flushing that accompanied our patient’s skin rash. From the massive release of mast cell mediators, our patient exhibited autonomic dysfunction with evidence of episodic tachycardia accompanied by generalized flushing and abdominal pain. Hannaford and Rogers conducted a retrospective case study of 173 children with mastocytosis, and flushing was reported in 100% of the cases with DCM, compared to 26% of UP cases, and 29% of mastocytomas [2].

Although the clinical picture of our patient was benign other than the systemic rash and episodic flushing and tachycardia, this is not always the case for patients with DCM. The massive release of mast cells can also lead to anaphylactic shock, especially in DCM as reported by Lange et al., where three of the ten patients experienced anaphylactic shock [9]. The massive release of mast cell mediators can even be fatal. A recent report describes a case of fatal DCM in a neonate with systemic involvement of the GI tract and associated malabsorption and hepatosplenomegaly [10]. A study by Murphy et al. also reported eight cases of fatal childhood mastocytosis in which DCM was more common [3].

Treatment of mastocytosis is based on symptoms, as most of the pediatric cases are known to resolve spontaneously by adolescence [11]. One of the mainstays of therapy includes H1 and H2 antihistamine drugs such as hydroxyzine and famotidine, which diminishes itching and gastric-acid secretion. Pimecrolimus cream and oral antihistamine together was found to inhibit calcineurin, further preventing the release of inflammatory cytokines and mediators from mast cells [12]. Mast cell stabilizers, such as cromolyn sodium and ketotifen, have been shown to benefit GI symptoms, pruritus, and flushing [13]. Phototherapy was found to demonstrate diminished dermatographism and disappearance of leathery skin thickening in DCM [14]. Corticosteroids have also been used to treat cutaneous mastocytosis. Rapid regression and reduced blistering bullous lesions were demonstrated when oral steroids were used. Treatment for more systemic symptoms includes biologics and monoclonal antibodies such as imatinib and omalizumab [14]. Chemotherapy continues to be discouraged unless there is an associated hematologic disease [15]. Furthermore, avoidance of triggers of mast cell release is important for these patients such as rubbing or scratching eliciting Darier's sign, heat or changes in temperature especially while bathing, spicy food or hot beverages, stress, dryness of the skin, exercise, insect bites, and medications (nonsteroidal anti-inflammatory drug (NSAID), morphine, and dextromethorphan). Our patient was initially sent home without any medications. However, during outpatient followup, the infant was started on hydroxyzine, famotidine, cromolyn sodium, and a short course of oral prednisolone for pruritus and persistent cutaneous lesions. Parental education was provided to avoid triggering factors and complications.

## Conclusions

This case of congenital DCM highlights a rare disease and the broad differential diagnoses that must be considered in the evaluation. Furthermore, we bring to light the importance of early biopsy and use of the biomarker tryptase when high suspicion of mast cell activation to establish a diagnosis which may save the patient numerous tests, invasive procedures, and unnecessary medications such as antibiotics. We emphasize the need to educate parents on the severe complications that could result from a massive release of mast cell mediators. Lastly, we list common triggers to avoid and review some of the treatments being used successfully in patients with DCM.
